# Insights into the Maturity of Keratin Byproduct-Based Biofertilizer by UV–Vis, Fourier Transform Infrared Spectroscopy, and Scanning Electron Microscopy Technologies

**DOI:** 10.1021/acsomega.6c00582

**Published:** 2026-05-14

**Authors:** Saloua Biyada, Eglė Marčiulaitienė, Renata Boris, Jurgita Malaiškienė, Jaunius Urbonavičius

**Affiliations:** † Civil Engineering Research Centre, 112678 Vilnius Gediminas Technical University, Saulėtekio av. 11, LT-10223 Vilnius, Lithuania; ‡ Department of Environmental Protection and Water Engineering, 112678 Vilnius Gediminas Technical University, Saulėtekio av. 11, LT-10223 Vilnius, Lithuania; § Institute of Building Materials, 112678 Vilnius Gediminas Technical University, Linkmenų str. 28, LT-08217 Vilnius, Lithuania; ∥ Department of Chemistry and Bioengineering, 112678 Vilnius Gediminas Technical University, Saulėtekio av. 11, LT-10223 Vilnius, Lithuania:

## Abstract

Dog hair keratin byproducts are extremely resistant to degradation and require appropriate waste management technologies. To enhance the degradability of these byproducts, silo cocomposting with food byproducts was performed for 19 weeks. In this study, the physical-chemical analysis (temperature, pH, electrical conductivity, carbon/nitrogen ratios (C/N), germination index, and mineral nutrients) was combined with spectroscopic technologies (ultraviolet–visible (UV–vis), Fourier transform infrared (FTIR) spectroscopy, and scanning electron microscopy (SEM)) as an advanced tool to effectively monitor the maturity of compost, thereby minimizing excessive consumption of time and products. Furthermore, a partial least-squares regression (PLS-R) model was used to predict the final compost quality. As a result, it can be concluded that the compost produced fulfills the norms of applicability of mature compost in agriculture with a C/N ratio below 20 and a germination index of 100%. These results were confirmed by UV-spectroscopy analysis showing an increase in humification and by FTIR depicting the degradation of disulfide bonds in cysteine present in keratin and the degradation of cellulose, hemicellulose, and lignin in food byproducts. This study confirms that a well-managed degradation of keratin byproducts enables them to be upgraded into a valuable product that could be used to improve the fertility of degraded soils and promote plant growth.

## Graphical abstract

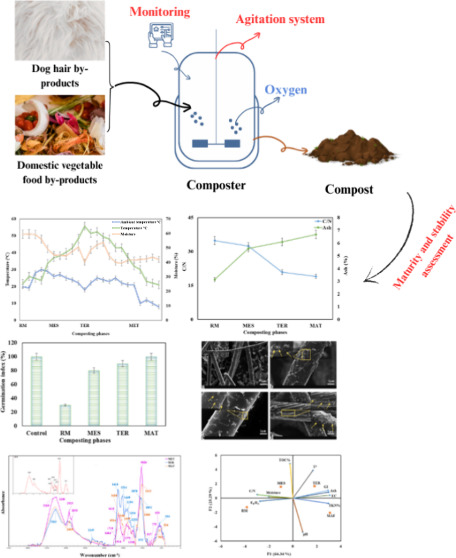

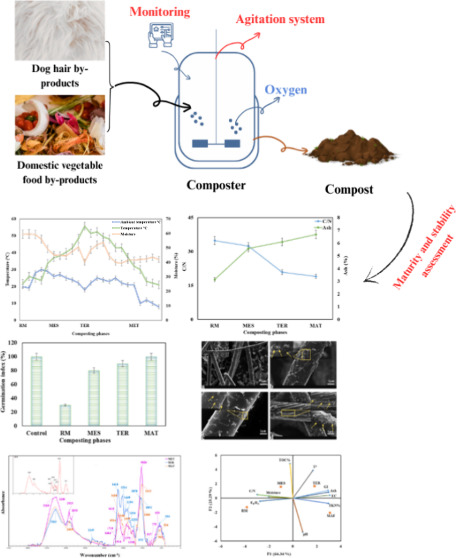

## 1 Introduction

Globally, millions of tons of keratin byproducts are discarded annually without being properly managed.^1^ Keratin remains the third most widespread polymer after cellulose and chitin.^2^ It belongs to the insoluble fibrous protein family, is extensively abundant in all biotopes, and is typically present in poultry feathers, horns, hooves, nails, wool, human hair, cat hair, and dog hair.^3^ The buildup of this byproduct in nature is considered a potential contributor to environmental contamination.^2,4^ However, these polymers can be reused for several purposes, including animal feed, biofertilizers, biofuels, and the biomedical field, after being biodegraded into soluble proteins, peptides, and amino acid residues.^1,5^ However, research on the upgrading of keratin byproducts (especially those derived from dog hair) into high-value-added products is still limited due to the strength and complexity of its structure, based mainly on multiple disulfide bridges between cysteine amino acids, as well as hydrogen and ionic bonds, making it difficult to solubilize under normal environmental conditions.^6^

Beyond their high cost, traditional methods of managing keratin byproducts, such as landfill, incineration, and chemical treatment, lead to a potential loss of valuable natural resources and further pollution.^7^ To overcome the challenges linked to the recalcitrant molecule degradation and the wastage of natural resources, a more sustainable approach is required. In this regard, microbial conversion of biomass has proven its effectiveness as an environmentally friendly process to recover a wide range of recalcitrant organic matter and turn it into high-value products.^3,5,8^ Composting is one of the most promising biological treatments. It harnesses the strength of microbial consortia and their enzymes to decompose recalcitrant molecules, particularly keratin, thereby producing organic fertilizers for agriculture while preserving their nutritional value.^3,7^ The composting environment allows the proliferation of mesophilic and thermophilic microorganisms (bacteria, actinomycetes, and fungi), which have an optimum growth temperature range between 20 and 60 °C. According to Fan et al.,^3^ thermal conditions improved keratin degradation and contributed to speeding up the process while reducing the risk of contamination, thereby making composting an appropriate solution for enhanced keratin byproducts' degradability while producing stable, mature compost. However, to guarantee the quality of the final compost, it is important to properly manage the composting process. In this regard, it is necessary not only to consider physical-chemical parameters but also to understand the changes in the surface structure and functional groups of the organic matter occurring throughout the composting in order to obtain mature and stable compost.

For this reason, the novelty of this study lies in the upgrading of keratin byproducts from dog hair through composting rather than closed-loop technologies, enabling the production of a high-quality biofertilizer that can then be applied to degraded soils, thereby supplying the required nutrients for plant growth. In this regard, the feasibility of keratin byproduct composting into a stable and mature biofertilizer was investigated using both traditional and advanced analysis. This research sought the development of a feasible strategy to further enhance the performance of dog hair byproduct composting mixed with food byproducts by controlling its degree of maturity using physical-chemical parameters, germination index, Fourier transform infrared spectroscopy, UV–visible spectroscopy, and scanning electron microscopy. Delving deeper into the process and using the PLS-R model, this study provides an insight into the correlation between quantitative and qualitative chemical properties achieved through the composting of dog hair byproducts, which can subsequently be used as a database for forthcoming research into the upgrading of keratin byproducts.

## 2 Materials and Methods

### 2.1 Feedstock Material Collection

The dog hair used in this study was collected from a dog-hair salon. This byproduct was manually sorted to remove all sorts of contaminants. The composition of the composting mixture is summarized in Table 1.

**Table 1:** Composting Mixture Composition

**main input waste**	**mixture for composting** (mass %)	**composting method**	**BP** (weeks)	**total** (weeks)
dog hair byproducts	dog hair 7% + domestic vegetable food byproducts 93%	silo composting	11	19

### 2.2 Experimental Design and Setup

The composting experiment was carried out on a commercial composter from **Oklin** (GC-02, Honkong, China, effective size 0.76 × 0.46 × 0.76 mL × W × H). The assembled materials were automatically overturned every day throughout the experiment to maintain aeration and dissipate the heat released during the thermophilic phase. The moisture content of the feedstock was regularly adjusted to 60% by sprinkling an adequate amount of tap water to provide sufficient moisture for the microorganisms to thrive. In order to obtain a composite and representative sample, the material was collected throughout the composter at different stages, from four cardinal positions (east, west, north, and south) and three depths (0–14 and 15–30 cm). The total composting process lasted for 19 weeks.

### 2.3 Experimental Analysis

#### 2.3.1 Physical-Chemical Analysis

Measurements of temperature, pH, electrical conductivity, and ash content were performed in conformity with the protocol described by the French Standardization Association (Afnor). The C/N ratio was calculated from the percentage of total organic carbon (TOC) and total Kjeldahl nitrogen (TKN) according to the protocol described by Afnor.^9^ The dried samples were finely ground and subsequently used for the measurement of total C and N. Chemical analysis of the compost samples was performed using X-ray fluorescence (Rigaku ZSX Primus IV, Japan). The following spectrometer parameters were used: Rh anode 4 kW, 60 kV; sample diameter 40 mm; and height 3 mm. The crushed materials were compressed with a strength of 200 kN for 3 min. The samples were tested in a vacuum environment (∼6 Pa) at a temperature of 36.5 °C.

Germination tests were conducted at room temperature (25 °C) in the dark for 72 h. This involved depositing 10 maize seeds on filter paper in Petri dishes soaked with 5 mL of water-soluble extracts of the compost (10 g/100 mL of distilled water) in different phases: raw material (RM); mesophilic phase (MES); thermophilic phase (TER); and maturation phase (MAT) in triplicate. The germination index (GI %) was calculated as the percentage of viability depending on emergence and root length in the water-soluble extract of each composting stage compared to the control.

#### 2.3.2 UV–Visible Spectroscopy Analysis

UV–visible spectroscopy was performed on compost extracts prepared by mixing 1 g of the sample from each phase (mesophilic phase (MES), thermophilic phase (TER), and maturation phase (MAT)) with 50 mL of NaOH (0.5 M). The extracts were shaken for 2 h, subsequently centrifuged (3000 rpm, 25 min), and the supernatant was filtered. UV–vis absorption (220–800 nm) was recorded at selected wavelengths (280, 472, and 664 nm), which allowed the absorbance ratios to be calculated. *E*_2_/*E*_6_ (OD_280_/OD_664_), *E*_4_/*E*_6_ (OD_472_/OD_664_), and *E*_2_/*E*_4_ (OD_280_/OD_472_) are intended to express the degree of humification.

#### 2.3.3 Scanning Electron Microscopy

Scanning electron microscopy (SEM) was performed by using a ThermoFisher Scientific Quattro S field emission scanning electron microscope (Eindhoven, Netherlands) to highlight changes in the microstructure of organic matter depending on the composting phase. Surface imaging was performed at a working distance of 10 mm and an accelerating voltage of 10 kV. Prior to imaging, the sample surfaces were coated with an electrically conductive gold layer (Au) by sputtering using a QUORUM Q150R ES instrument (Laughton, UK).

#### 2.3.4 Fourier Transform Infrared Spectroscopy Analysis

Changes in functional groups throughout composting treatment were determined in different phases of treatment ((MES), thermophilic phase (TER), and maturation phase (MAT)) and in raw materials (RM) before composting occurred, using a Fourier transform infrared (FTIR) spectrometer (Invenio R, Bruker, Billerica, MA, USA). Spectral data were collected in the range of 400–4000 cm^–1^.

#### 2.3.5 Statistical Analysis

In order to evaluate the quality of the compost obtained from the dog hair byproduct, the physical-chemical fractions were statistically analyzed. The mean, standard deviation (St. Dev.), and coefficient of variation (CV) were determined. Furthermore, a standardized variance analysis (one-way ANOVA) was used to compare the means. Partial least-squares regression (PLS-R) was performed to correlate the chemical fractions of the compost samples with the UV–vis and FTIR spectra.

## 3 Results and Discussion

### 3.1 Physical-Chemical Analysis

A detailed description of the raw materials used in this study is provided in Table 2. Dog hair is characterized by a high nitrogen content (14.3%) and a low C/N ratio (close to 4%). The low C/N ratio is mainly due to the high nitrogen content of the dog hair. On the basis of preliminary results and in order to increase the carbon content of the mixture, food byproducts are added. These byproducts are characterized by a high carbon content (51.9%), moisture content (96.3%), C/N ratio (47.2), and a low nitrogen content (1.1%). The combination of dog hair and food byproducts provides an initial mixture with a C/N ratio of 35, which meets the standard for suitable composting raw materials.

**Table 2:** Physical-Chemical Characterization of the Feedstock^a^

**physical-chemical parameters**	**dog hair**	**food waste**	**norm** NFU44–051/A2
**moisture %**	90.22 ± 1.3	96.25 ± 1.26	40–60
**pH**	6.70 ± 0.84	5.60 ± 0.15	6.5–8.5
**EC (mS/cm)**	0.32 ± 0.03	4.65 ± 0.35	
**total carbon (TC %)**	48.1 ± 0.09	51.85 ± 1.30	>20
**total nitrogen** (TN %)	14.3 ± 0.07	1.1 ± 0.06	
**C/N ratio**	3.4	47.15	20–40

a Values designate mean ± standard deviation based on 3 samples.

Physical-chemical changes in the compost are monitored in different phases (mesophilic phase (MES), thermophilic phase (TER), and maturation phase (MAT)). Figure 1 illustrates the temperature changes that occurred during composting.

**Figure 1:**
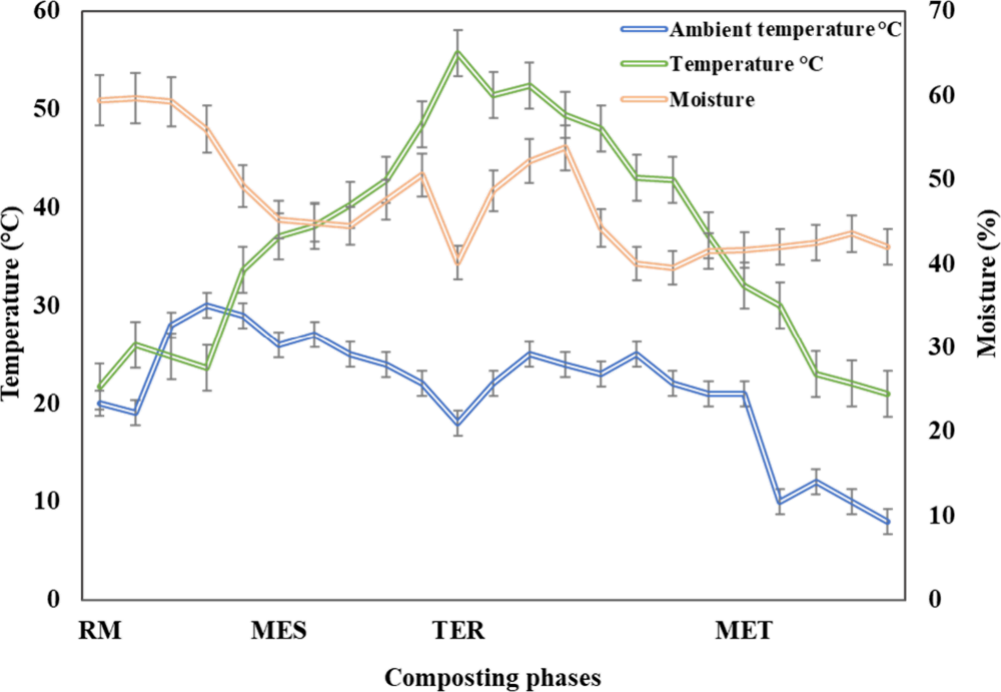
Temperature profile and moisture evolution throughout the different phases of cocomposting (raw material, RM; mesophilic phase, MES; thermophilic phase, TER; and maturation phase, MAT) of dog hair and food byproducts.

The temperature curves show the typical behavior of the composting process, with an increase in temperature followed by a decrease at the end. In this regard, from the beginning of composting, an increase in temperature was recorded, particularly during the thermophilic phase (above 56 °C, after 11 weeks), exceeding the ambient temperature. This increase is generally due to the microbial metabolism of organic matter, which generates heat during composting and therefore an increase in temperature.^10^ The thermophilic phase lasted 3 weeks, confirming that the compost produced is safe from pathogens. In the maturity stage of composting, a decrease in temperature was reported, potentially related to mechanical turning and a decrease in microbial activity.^11^ Toward the end, the temperature gradually tends to stabilize, confirming the maturity and stability of the compost produced. Regarding moisture changes during composting, it should be noted that the moisture graph is linked to temperature changes (Figure 1). At the beginning, the humidity was around 50%, reflecting typical conditions for microbial growth. A decrease in humidity was recorded simultaneously with an increase in temperature, leading to the evaporation of water.^12^

Figure 2 shows the changes in pH and conductivity during the cocomposting of dog hair and the food byproduct. The pH fluctuations play a pivotal role in determining microbial activity and consequently in the transformation and humification of organic matter.^10^ In this regard, a decrease was noticed at the beginning of composting, which could be related to the degradation of readily degradable organic matter by microorganisms into organic acids and CO_2_, particularly polysaccharide compounds contained in food byproducts.^13^ During the thermophilic phase, pH shifts toward alkalinity, probably linked to ammonia production resulting from keratin degradation by microorganisms. The solubilization of ammonia leads to the formation of ammonium and thus to an increase in pH values.^14^

**Figure 2:**
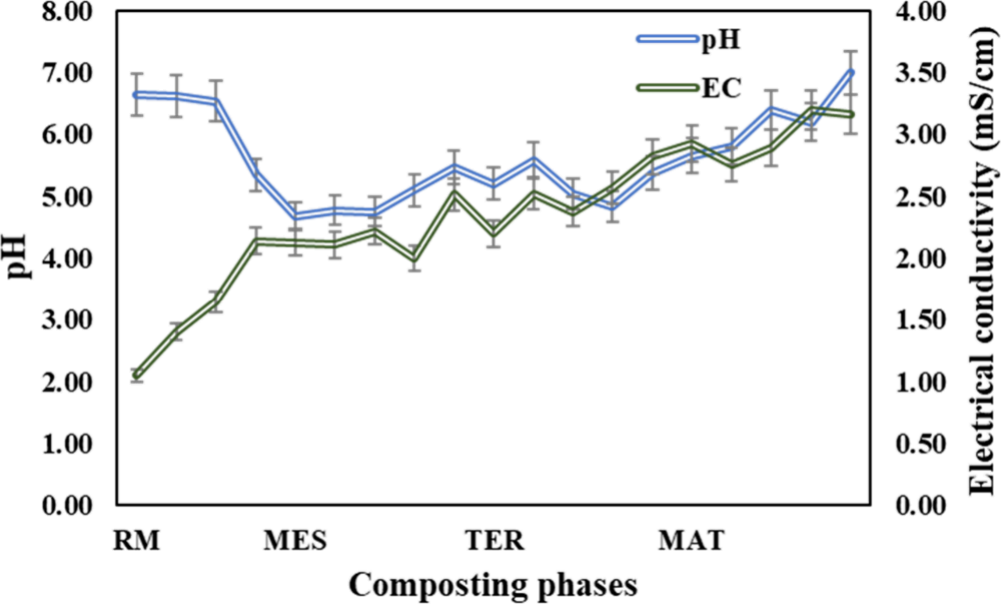
Changes in pH and electrical conductivity throughout the different phases of cocomposting (raw material, RM; mesophilic phase, MES; thermophilic phase, TER; and maturation phase, MAT) of dog hair waste and food waste.

The higher electrical conductivity (3.2 mS/cm) recorded during composting was apparently attributed to organic carbon degradation (Figure 2), releasing hydroxyl ions through ligand exchange and ions such as phosphates and ammonium. In this regard, according to Abul Hashem et al.,^13^ a final compost with an electrical conductivity below 4 mS/cm is considered to be bearable for plants.

Although dog hair has a low C/N ratio due to its high nitrogen content, supplementing the mixture with food byproduct increases this ratio to reach an acceptable level for starting, which is usually around 30. The C/N ratio plays a significant role in the overall composting process as the final results depend on the initial C/N ratio of the mixed materials.

From the first weeks of treatment, a slight decrease in the C/N ratio was recorded, which was more pronounced during the thermophilic phases, followed by stabilization during the maturation phase at C/N = 19 (Figure 3). Comparable results have been achieved with the cocomposting of other high-nitrogen residues, such as poultry feathers.^15^ The decrease in the C/N ratio directly linked to the decrease in carbon content results from organic matter mineralization, leading to an increase in nitrogen levels over time, and this is further confirmed by the increase in ash levels, which is directly linked to the process of organic matter mineralization and, consequently, to increased mineral content,^16^ (see also Figure 3). In fact, the C/N ratio has been identified as an indicator of the final compost stability; a ratio between 15 and 20 indicates mature and stable compost that can be used as an agricultural fertilizer.^17^

**Figure 3:**
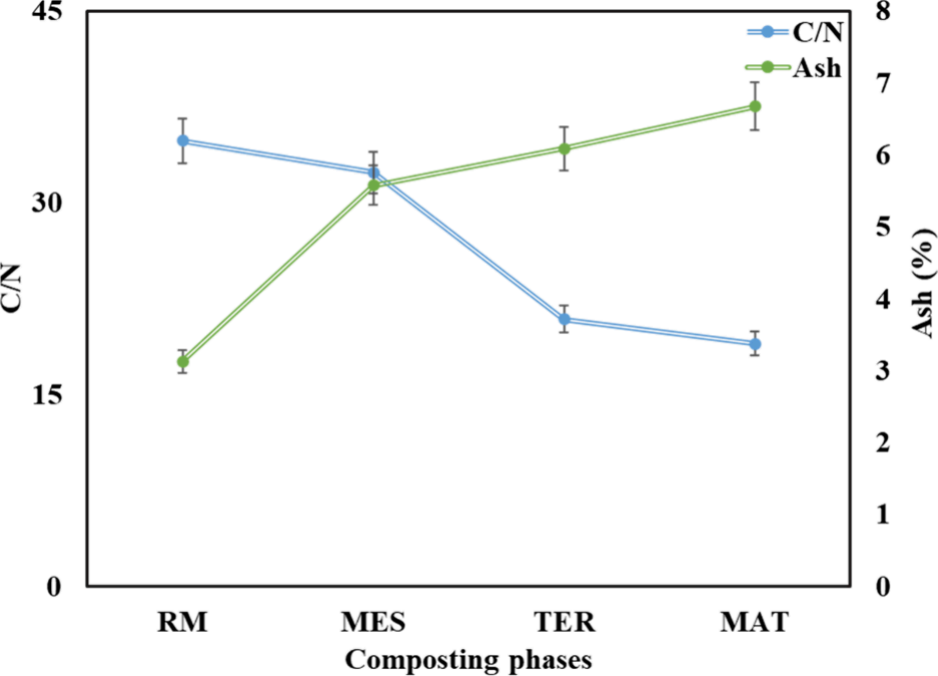
Evolution of the C/N ratio and the ash content according to the composting phases (raw material, RM; mesophilic phase, MES; thermophilic phase, TER; and maturation phase, MAT) of dog hair waste and food waste.

Figure 4 illustrates the behavior of the oligoelement content throughout the composting phases. From the outset, a considerable increase in the nutrient content (Na^+^, P, and K^+^) was recorded. It is noteworthy that the increase in micronutrients could be linked to mineralization and/or humification of organic matter by microorganisms throughout the composting process.^18^ This result proves that the final compost produced from dog hair and food byproducts is a source of nutrients and fertilizers that have a pivotal impact on plant growth, boosting their vegetative activity and germination and, therefore, seed growth.

**Figure 4:**
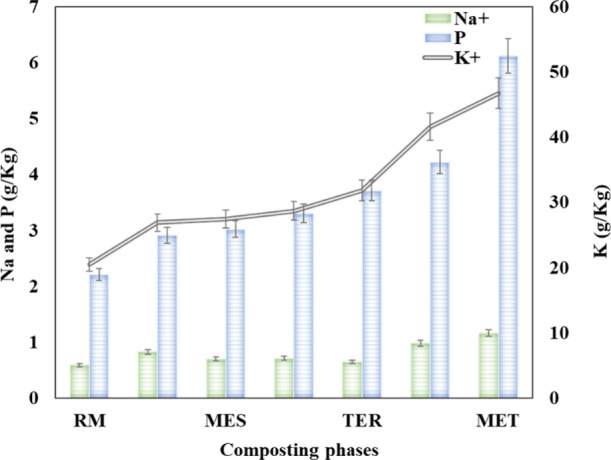
Evolution of mineral nutrients according to composting phases (raw material, RM; mesophilic phase, MES; thermophilic phase, TER; and maturation phase, MAT) of dog hair and food byproducts.

In order to ensure that the compost produced is nontoxic, a germination test was successfully conducted. Figure 5 reveals that the initial mixture (RM) has a phytotoxic effect on corn seeds, with a germination index considerably lower than 50% (30%), which is fairly typical for untreated materials. In fact, the low germination index recorded could be linked to the inaccessibility, buildup of germination inhibitor molecules (such as phenolic compounds, alcohols, and organic acids), and/or low concentration of oligoelement nutrients in the initial mixture,^19^ which has already been confirmed by mineral nutrient analysis (Figure 4). Throughout the composting process, the mixture used exhibited an increase in GI from the mesophilic phase of composting onward. Ultimately, the compost produced had a GI of 100%, comparable to that of the control test, confirming its phytonutritive and phytostimulant benefits for plant growth. These results are consistent with those obtained by Siles-Castellano et al.^20^

**Figure 5:**
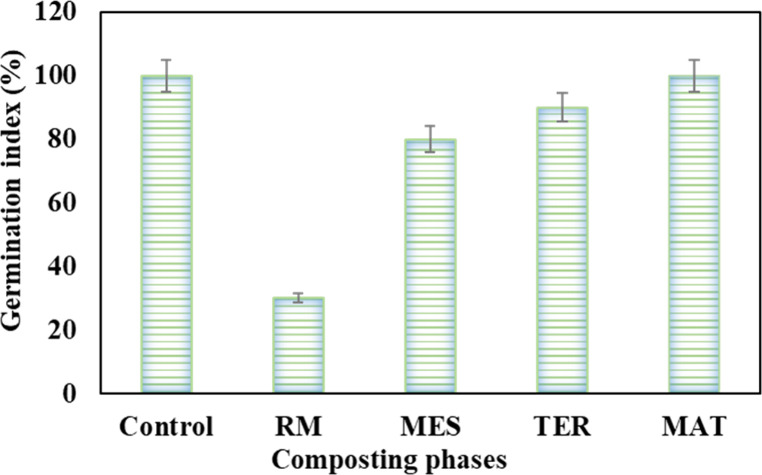
Evolution of germination index of corn seeds during composting phases (raw material, RM; mesophilic phase, MES; thermophilic phase, TER, and maturation phase (MAT)) as well as in the control.

### 3.2 UV–Visible Spectroscopy Analysis

UV–visible spectroscopy data enables in-depth insight into the transformation and humification of biomass during composting. It is widely considered to evaluate the degree of maturation based on the following absorbance ratios: *E*_2_/*E*_4_ = λ_280_/λ_472_, *E*_2_/*E*_6_ = λ_280_/λ_664_, and *E*_4_/*E*_6_ = λ_472_/λ_664_, since the majority of humic substances absorb light significantly at these wavelengths. This was confirmed by Hanc et al.,^21^ revealing that absorption in this UV–vis region (200–700 nm) involved an overwhelming predominance of chromophores containing aromatic rings with different degrees and types of substitution, such as monosubstituted and polysubstituted phenolic compounds and a wide range of monoaromatic and/or polyaromatic acids. Table[Table tbl3] reveals a significant decrease in all ratios from week 4 onward, followed by stabilization around week 9 and a slight decrease at the end of treatment, with values of 24.24, 4.30, and 5.93, respectively, for the *E*_2_/*E*_6_ ratio, the *E*_4_/*E*_6_ ratio, and the *E*_2_/*E*_4_ ratio. In this regard, the decrease in absorbance rates at the beginning could be related to the decomposition of readily degradable organic matter, particularly derived from food byproducts, which is likely to be swiftly degraded by microorganisms.^22^ In the current study, food byproducts were used to provide microorganisms with an accessible source of carbon and energy for their growth to produce the essential enzymes for the degradation of high molecular weight compounds, in this case, keratin.^23^ Furthermore, the stability recorded in these ratios in the ninth week could be referred to as a remarkable increase in phenolic compounds, which form essential components of humic substances. The slight decrease observed at the end of the composting, particularly for the *E*_4_/*E*_6_ ratio, revealed the development of humified matter. The *E*_4_/*E*_6_ ratio is commonly used as a humification index that describes the degree of aromatization and polymerization of organic compounds. These results are consistent with those achieved by Hanc et al.; Li et al.^21,22^

**Table 3:** Evolution of Absorbance of Compost Samples during Composting

**time** (weeks)	***E***_**2**_ **/*E***_**6**_	***E***_**4**_ **/*E***_**6**_	***E***_**2**_ **/*E***_**4**_
1	46.82	5.64	8.30
4	37.13	5.56	6.67
7	32.04	4.44	7.21
9	29.66	4.66	6.37
13	24.24	4.30	6.33
19	28.97	4.88	5.93

### 3.3 Scanning Electron Microscopy

Changes in the microstructure of the compost samples were evaluated throughout the different phases of composting (Figure 6b–d) (see also the control in Figure 6a) using a scanning electron microscope (SEM). Studies have revealed structural changes in the samples. The control samples preserved their original compact and smooth surfaces (Figure 6a). Nevertheless, deterioration of dog hair is clearly visible, notably in the advanced composting phases. Cracks, grooves, and the consequent loss of successive layers of the sample surface were observed. These results are in line with those achieved by Broda et al.^24^ during the biodegradation of sheep’s wool and Abul Hashem et al.^13^ during the composting of different types of keratin byproducts. The changes that occurred on the surface of dog hair during composting are mainly due to microbial metabolic activity. In this regard, several studies have attributed surface changes during composting to microbial enzymes, notably keratinases, proteases, cellulases, etc.^25–28^

**Figure 6:**
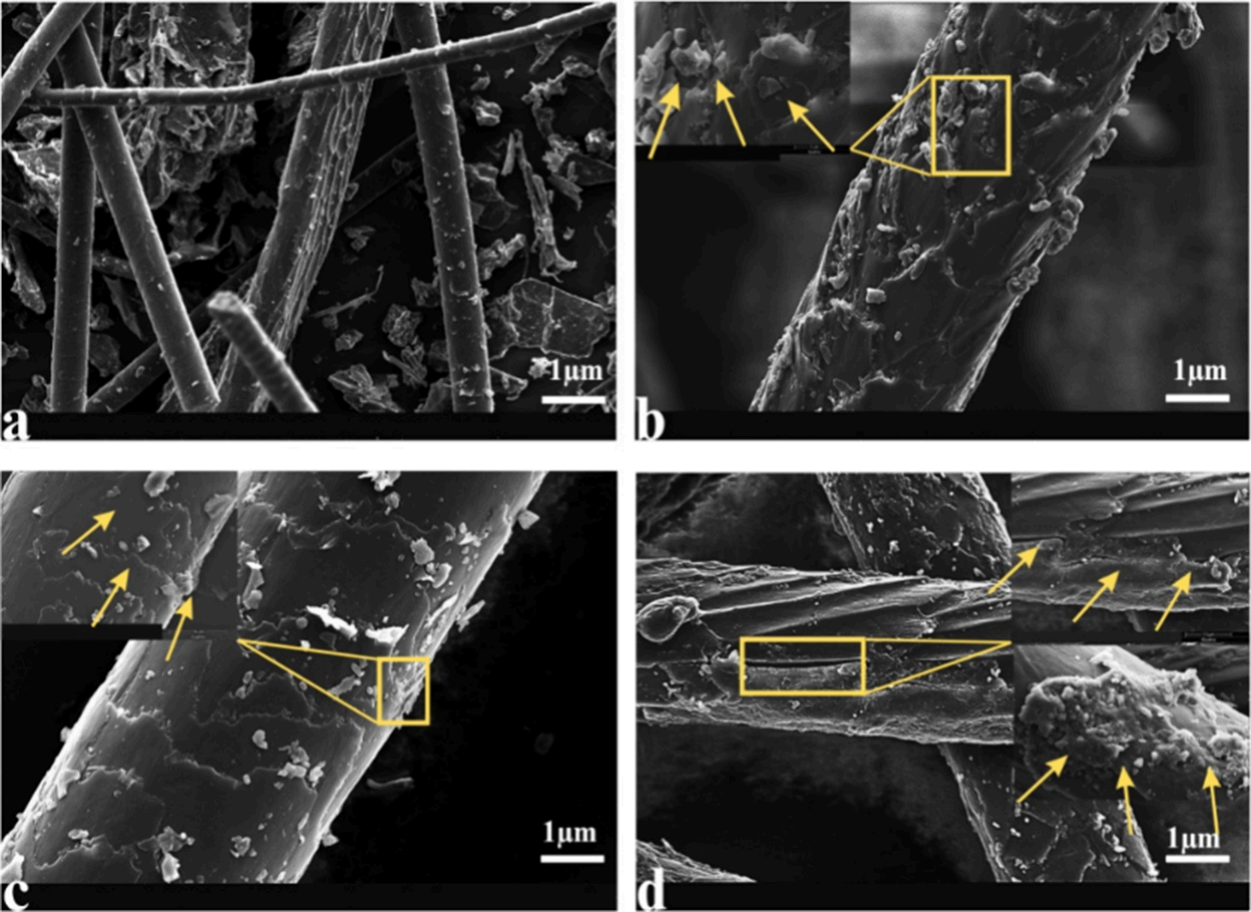
SEM images of dog hair surfaces during composting phases (a-raw material; magnification: *2500; b-mesophilic phase; magnification: *3500; c-thermophilic phase; magnification: *30,000, and d-maturation phase). The yellow arrows indicate the alterations wrought on the dog hair surface by the microorganisms during the test period.

### 3.4 Fourier Transform Infrared Spectroscopy Analysis

FTIR spectra of samples before (RM) and during the different phases of composting (MET, TER, and MAT) are presented in Figure 7. The spectrum reveals several distinct broad bands, reflecting the abundance of aromatic compounds, phenolic, peptide, aliphatic, and polysaccharide structures, in the mixture tested.

**Figure 7:**
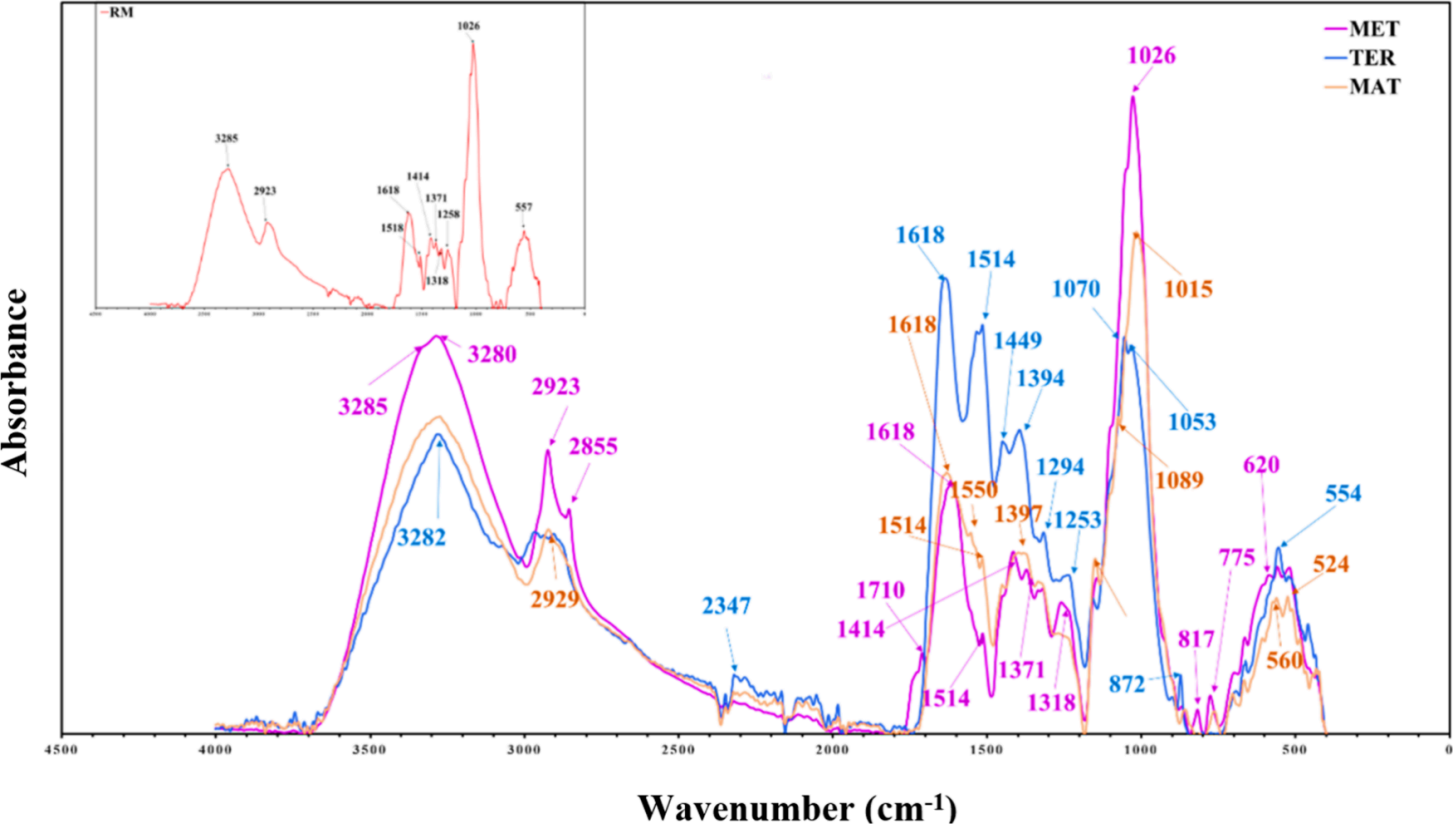
FTIR spectra of samples from different composting phases (RM, MES, TER, and MAT).

In this regard, bands ranging from 3300 to 3000 cm^–1^ are ascribed to OH stretching involving hydrogen in the water present in keratin and crystalline cellulose as well as in hemicelluloses and lignin, while those located within the 3000–2700 cm^–1^ region are assigned to stretching of lipid alkyl chains (methyl and ethyl).^24,27,29^ The band at 2900 cm^–1^ is mainly linked to the stretching vibration of the methylene (−CH_2_−) bond in the cellulose structure. It is clear that the typical absorption bands in the infrared spectrum are predominantly due to the absorption of energy by peptide bonds, which are specific to the structures of proteins. In fact, keratin’s major structural chain units are identified as amides I, II, and III. In this regard, amide I is associated with C–O stretching and appears at 1700–1600 cm^–1^, while amide II is linked to N–H bending and C–H stretching vibration and is located in the range 1540–1520 cm^–1^, whereas amide III is predominantly linked to a combination of N vibration, C–N stretching vibration, and C–O bending vibration and is located between 1220 and 1300 cm^–1^.^24,30^ It is noteworthy that in the raw material sample, the bands linked to the peptide bonds were clearly distinguishable and exhibited high intensity, and the amide I, II, and III bands were conspicuous at 1618, 1518, and 1258 cm^–1^, respectively. These results are consistent with those reported in several studies on keratin waste.^24,27^ Moreover, the typical peak near 1550 cm^–1^ is attributed to the stretching vibration of the dehydrated glucose ring −C–O bond in the cellulose −C–O–C group.^31^ The band at 1029 cm^–1^ is indicative of the C–O–C bond vibration of the β-glycosidic linkages between glucose molecules in cellulose, predominantly indicating the presence of cellulose.^32^ Furthermore, the peak at 1399 cm^–1^ has been reported to be attributable to mineral forms such as ammonium bicarbonate formed by ammonia and CO_2_ released during composting. Significant absorption bands are detected in the ranges 600–620 and 500–550 cm^–1^ and are attributed to sulfide S–S bonds and sulfur bonds in cysteine C–C and C–S, respectively.^24^

During the biodegradation process, almost all bands are affected by microorganisms, mainly due to the action of microbial enzymes released, which disrupted the entire structure of the organic matter in the tested mixture. This can be clearly noticed from the mesophilic phase onward, through changes in intensity as well as the disappearance and appearance of bands. The keratin breakdown pathway is triggered by the breakdown of disulfide bonds in cysteine. Once these bonds are broken, keratin remains highly vulnerable to keratinolytic enzymes.^24^ The cleavage of the disulfide bond in cysteine is a sign of the onset of biodegradation, which is reflected by the appearance of a band at 1089 cm^–1^ attributed to the S–O bond associated with the transformation of cysteine into cysteic acid, which is mainly due to the microbial action. Oxidized forms of sulfur play a significant role in the oxidation pathway, a multistep process that leads to cysteic acid formation.^27^ Keratin peptide bonds were also significantly affected during composting, and their position mostly changed. The amide I peak shifted to higher values, increasing from 1618 cm^–1^ (RM sample) to approximately 1648 cm^–1^ (at the maturation stage). The same pattern was noted for amide II: moving from 1518 cm^–1^ to around 1550 cm^–1^, and for amide III, upward from 1258 cm^–1^ to around 1294 cm^–1^. These results are consistent with those achieved by Ramya et al.; Călin et al.; Broda et al.,^24,27,33^ in which keratin waste from sheep wool and horse hair was used as raw material for a biodegradation test. Cellulose, hemicellulose, and lignocellulosic compounds were also affected by microorganisms during composting. Indeed, the intensity of the peaks around 1420 cm^–1^ increased, potentially linked to the steady degradation of cellulose-rich compounds leading to the condensation of these structures within the humic complex,^29,31^ thereby revealing an increase in the degree of polymerization. These results are also confirmed by the UV–vis analysis. Additionally, a disappearance of the band at 817 cm^–1^ was recorded, potentially reflecting the disruption of C–O–C bonds related to crystalline cellulose I in the β-(1–4) glycosidic bond, mainly caused by microorganisms,^29,31,34^ which is confirmed by the disappearance of the band at 1500 cm^–1^ in the maturation stage, demonstrating the breakdown of lignocellulosic components. Ultimately, these results highlighted noticeable and conspicuous changes in peak intensity toward the thermophilic phase, pointing to significant conformational changes in the analyzed samples, mainly due to the degradation of peptide, polysaccharide, and lignocellulosic components triggered by microorganism enzyme activity. FTIR results reinforce the outcome of UV–vis and chemical analyses.

### 3.5 Statistical Analysis

The factorial map illustrated the samples collected from different composting phases, as indicated by their barycentric coordinates. F1 and F2 covered 64.34 and 25.29% of the variance in the spectral data, respectively (Figure 8).

**Figure 8:**
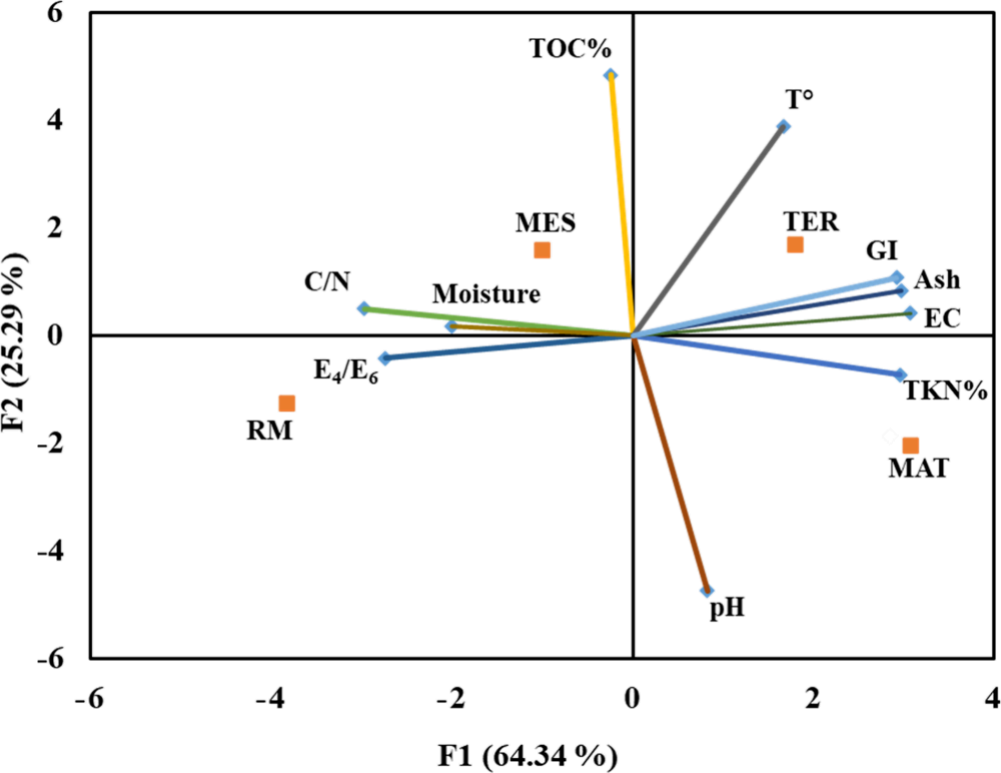
Results of PCA analysis. Each circle is the barycenter coordinate with the standard deviation of each composting phase.

The principal component analysis (PCA) confirms that the physical-chemical parameters of the composts evolved throughout the different phases of composting. A chronological distribution of samples on the F2 was recorded. C/N ratio, C, N, *E*_4_/*E*_6_ ratio, ash, pH, temperature, and moisture content: arrows refer to mature compost (MAT) and fresh compost (RM). This analysis explains the results obtained by physical-chemical and spectral analysis on the stability and maturity of the compost from keratin byproducts; the C/N and *E*_4_/*E*_6_ arrows, directed from mature compost to fresh compost, show the organic matter stabilization and strengthen the chronological distribution in F2.

The purpose of this study was to determine the most appropriate conditions for the production of mature compost from keratin byproducts. For this reason, organic matter status prediction based on time using infrared analysis was applied to predict the physical-chemical parameters. The reliability of the prediction model was evaluated by using several parameters (Table 4).

**Table 4:** Summary Statistics of Compost Analytical Parameters, UV–Visible, and Infrared Spectra throughout Different Composting Phases^a^

	**main**	St. Dev.	**CV** (%)^a^	**ANOVA**	***R*** ^**2**^	RMSEP^a^
CT^a^	7.75	5.9	21.72	<0.05	0.66	4.21
TOC (%)	44.20	0.55	43.97	<0.05	0.05	0.40
TN (%)	1.77	0.52	2.77	0.20 ng	0.93	0.11
C/N	26.70	7.99	11.53	<0.05	0.93	1.76
*T* (°C)	36.56	14.43	52.13	<0.05	0.29	10.46
moisture	50.14	7.78	47.07	<0.05	0.03	6.68
pH	6.01	0.76	6.48	0.69 ng	0.09	0.63
EC (mS/cm)	2.29	0.93	3.79	0.24 ng	0.66	0.46
ash	5.44	1.26	7.22	0.60 ng	0.50	0.76
IG (%)	75.00	24.58	117.14	<0.05	0.47	19.50
*E*_4_/*E*_6_	5.18	0.48	4.36	0.56 ng	0.96	0.07

a CV: coefficient of variation; significance levels: ns: not significant; *p* < 0.05; *R* ^2^: coefficient of correlation; RMSEP: root-mean-square error of prediction.

The coefficient of correlation (*R* ^2^) values were 0.05, 0.93, 0.93, 0.29, 0.03, 0.09, 0.66, 0.50, 0.47, and 0.96, respectively, for TOC, TN, C/N, *T*°, moisture, pH, EC, ash, IG, and *E*_4_/*E*_6_ ratio. The values obtained from this analysis demonstrate that these parameters correlate well with the IR spectra. The RMSEP values were 0.4, 0.11, 1.76, 10.46, 6.68, 0.63, 0.46, 0.76, 19.50, and 0.07, respectively, for TOC, TN, C/N, *T*°, moisture, pH, EC, Ash, IG, and *E*_4_/*E*_6_ ratio. The low values obtained by RMSEP indicate an acceptable correlation between these parameters and the IF spectra. Regarding the coefficient of variation (CV %), the values were 43.97, 2.77, 11.53, 52.13, 47.07, 6.48, 3.79, 7.22, 117.14, and 4.36, respectively, for TOC, TN, C/N, *T*°, moisture, pH, EC, Ash, IG, and *E*_4_/*E*_6_ ratio. An ANOVA test was used to depict the changes of the physical-chemical properties throughout composting phases. Significant differences (*p*-value < 0.05) were recorded for most of the parameters except TN, pH, EC, ash, GI, and *E*_4_/*E*_6_ ratio. PLS-R prediction between physicochemical parameters, UV–visible, and IR spectra reveals that they are well correlated, explaining the intensity changes of the different infrared peaks detected. These results were also confirmed using Pearson’s correlation coefficients (Table 5).

**Table 5:**
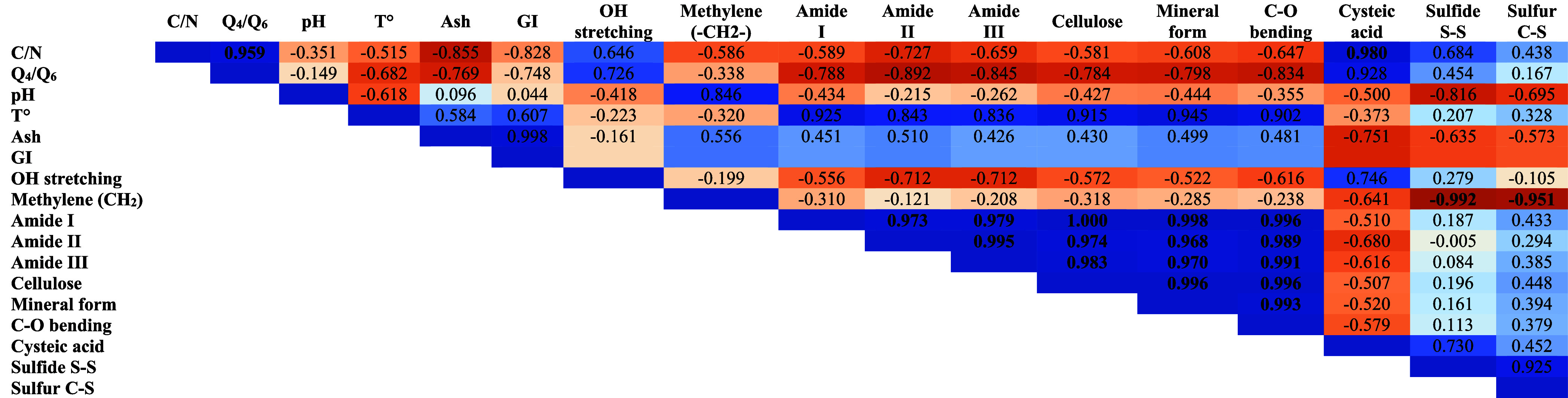
Pearson Correlation Coefficients Matrix between Physical-Chemical Parameters, UV–Visible, and Infrared Spectra^a^

a The blue color depicts positive correlation; the red color depicts negative correlation.

Pearson correlation coefficients were used to establish the possible correlation between key factors, physical-chemical properties, germination index, and spectral analysis of compost throughout the different composting phases. Table 5 shows that most of the physical-chemical parameters are strongly correlated. It is noteworthy that the C/N ratio was negatively correlated with nitrogen compounds and the germination index, while a positive correlation with carbon compounds was observed. This was unlike temperature, which was positively correlated with the nitrogen compounds and the germination index and negatively correlated with carbon compounds. Ultimately, statistical analysis confirms that although keratin byproducts are recalcitrant and barely degradable, the mixture used in this study produces stable and mature compost. It depicts a useful model for keratin byproduct upgrading.

## 4 Conclusions

Keratin byproducts are notoriously persistent, making them a major concern for the environment. This work confirmed that cocomposting dog hair with food byproducts can be a sustainable and environmentally friendly approach to upgrading these byproducts into the biofertilizer. In this regard, the success of the process is directly linked to the appropriate conditions and well-managed treatment. The device used for keratin byproduct composting allows for the production of a final compost with a C/N ratio of less than 20 and a germination index of 100%, demonstrating its maturity. This result was confirmed by UV–vis spectroscopy analyses, which revealed that there was an increase in the degree of humification. Also, SEM micrographs displayed characteristic degradation of keratin fibers throughout the composting phases, with multiple cracks and grooves on the surface. On the other hand, FTIR analysis highlighted a decrease in aliphatic but not aromatic compounds, with a breakdown of disulfide bonds in cysteine, proving the degradation of dog hair by microorganisms. The statistical model used in this analysis demonstrated that the use of a well-managed cocomposting system for the treatment of keratin byproducts enabled the production of mature and stable compost.
